# Slow wave sleep as a biomarker and therapeutic target for Alzheimer's disease

**DOI:** 10.1002/alz70856_101081

**Published:** 2025-12-24

**Authors:** Brianne A Kent, Mayuko Arai

**Affiliations:** ^1^ Simon Fraser University, Vancouver, BC, Canada

## Abstract

**Background:**

Sleep disturbances are prevalent among individuals diagnosed with Alzheimer's disease (AD), typically manifested as low levels of slow‐wave sleep (SWS; N3 stage). Restoring SWS has been proposed as an intervention to slow AD progression. Trazodone, an antidepressant with sleep‐promoting properties, is frequently prescribed off‐label to improve sleep. Notably, it is well‐tolerated in elderly individuals and has been shown to specifically enhance SWS. Given its potential benefits, trazodone has been proposed as an intervention for AD. However, research on its effects beyond its antidepressant properties remains limited. This study aimed to evaluate the effects of chronic trazodone administration in a mouse model of AD, marking the first assessment of its potential as a disease‐modifying therapeutic for AD.

**Method:**

We have developed a translationally‐focused administration paradigm for trazodone in C57BL/6J mice and demonstrated that acute trazodone administration dose‐dependently enhances NREM sleep and increases delta power during NREM sleep. Using this validated protocol, we administered trazodone (60mg/kg) daily to APP^NL‐F^ mice (up to 60 days) to evaluate its effects on EEG power spectra, behaviour/cognition, and neuropathology. Two age groups were selected to represent early (9 months) and intermediate (14 months) stages of neuropathology.

**Result:**

Preliminary analysis revealed that trazodone consistently enhances slow oscillation and delta power during NREM sleep over a 60‐day treatment period (*p* = 0.0040, comparing baseline with 1, 4, and 8 weeks of treatment in the 9‐month‐old cohort). Importantly, preliminary neuropathological analysis reveals that 60 days of trazodone treatment leads to lower levels of Aβ pathologies, particularly in male mice. Specifically, in male APP^NL‐F^ mice treated with trazodone from 14 to 16 months of age, the treatment was associated with significantly lower amyloid plaques in the dorsal hippocampus (46 % lower, *p* =  0.0174) and fibrillary plaques in the parietal cortex (18% lower, *p* =  0.0495). Furthermore, trazodone treatment in the male mice showed a downward trend in insoluble Aβ_42_ levels (40% lower, *p* =  0.0555). Touchscreen cognitive testing is ongoing in 13‐ to 14‐month‐old APP^NL‐F^ mice.

**Conclusion:**

Our research suggests that trazodone should be explored as a potential disease‐modifying therapeutic for AD.

